# Segmental Shielding: A Rare Case of Acute Respiratory Distress Syndrome with Middle Lobe Sparing

**DOI:** 10.7759/cureus.57985

**Published:** 2024-04-10

**Authors:** Faryal Altaf, Vedangkumar Bhatt, Mohit Sekhon, Shitij Shrivastava, Naqash Mazhar, Sarah Moore

**Affiliations:** 1 Internal Medicine, BronxCare Health System, New York, USA; 2 Medicine and Surgery, BronxCare Health System, New York, USA; 3 Medicine, California Institute of Behavioral Neurosciences & Psychology, Fairfield, USA; 4 Obstetrics and Gynecology, American University of the Caribbean School of Medicine, Cupecoy, SXM

**Keywords:** diagnostic challenge, mechanical ventilation, critical care, pulmonary vascular permeability, hypoxemia, atypical presentation, middle lobe, lobar sparing, ards, acute respiratory distress syndrome

## Abstract

Acute respiratory distress syndrome (ARDS) presents a formidable challenge in critical care, often resulting in high mortality rates, particularly in severe cases or those compounded by preexisting conditions. Despite substantial advancements in critical care, the heterogeneous nature of ARDS necessitates nuanced clinical approaches. ARDS is generally diagnosed through clinical evaluation, radiographic imaging, and laboratory tests, as well as acute onset, bilateral lung infiltrates on imaging, and a partial pressure of oxygen in arterial blood (PaO2)/fraction of inspiratory oxygen concentration (FiO2) ratio of less than 300 mmHg. Management involves measurements to improve oxygenation and provide mechanical ventilation to assist breathing. The typical manifestation of ARDS is diffuse lung involvement, which affects multiple lobes symmetrically. Here, we report an unusual case of ARDS in a 53-year-old female who was brought into the hospital in an unresponsive state, exhibiting hypoxic and hypotension requiring intubation. Subsequent imaging revealed a distinctive pattern: the preservation of the right middle lobe, diverging from the conventional diffuse pulmonary affliction. This case underscores the need for clinical vigilance and adaptability, as such atypical presentations can confound diagnosis and management, posing unique clinical challenges. This case highlights the importance of recognizing ARDS' diverse presentations. Moreover, understanding the mechanisms behind the lobar sparing could provide greater insight into the disease heterogeneity and guide tailored therapeutic approaches. The imperative for further research into these uncommon presentations is clear, as it may be vital to improving outcomes for a broader spectrum of ARDS patients.

## Introduction

ARDS represents a dysregulated and exaggerated inflammatory response in the lungs; it involves a complex interplay of inflammatory mediators, endothelial dysfunction, alveolar epithelial injury, and disrupted repair mechanisms [1]. It is characterized as noncardiogenic pulmonary edema and thus necessitates thorough evaluation to differentiate it from cardiogenic pulmonary edema and other potential causes of acute hypoxic respiratory failure [1]. Generally, ARDS arises from diffuse inflammatory lung injury triggered by various factors such as pneumonia, pancreatitis, sepsis, aspiration, and blood transfusion reaction [1]. Inflammatory cells release many inflammatory mediators, including Interleukin (IL)-6, IL-1, and tumor necrosis factor (TNF)-alpha [2]. This cascades into damage to the endothelial cells, which line the pulmonary capillaries, causing vascular permeability. Pulmonary edema and impairment of gas exchange occur upon leakage of protein-rich fluid and inflammatory cells into the alveolar spaces [2]. Further, sequelae of the protein-rich fluid into the alveoli cause the formation of hyaline membranes and further gas exchange impairment by increasing the diffusion distance for oxygen and carbon dioxide [3,4]. The inflammatory response of ARDS further activates the coagulation cascade, causing microvascular thrombosis and increasingly impairing pulmonary perfusion [3].

Moreover, dysregulated repair mechanisms can result in excessive fibroblast activity and the development of fibrotic tissue within the lungs, contributing to long-term complications such as pulmonary fibrosis [4,5]. In many cases, the inflammatory response would resolve and allow for restoration of lung function; however, in severe cases, ARDS would progress to irreversible lung injury and respiratory failure, and hence, patients would require mechanical ventilation [5-7]. It is important to note that ARDS can extend beyond pulmonary involvement and can progress to systemic effects such as cardiovascular dysfunction, renal failure, and multiorgan failure, which can significantly complicate the clinical course and prognosis [7,8]. 

## Case presentation

A 53-year-old female with a past medical history of hypertension, diabetes mellitus, alcohol use disorder, and hyperlipidemia presented with dizziness. She was found with altered mental status in a nursing home and was brought to the emergency department (ED) for further management. On her way to the ED, she had an episode of seizure. She was cyanotic with blood gas showing pH of 6.25, partial pressure of carbon dioxide (PCO2) of 63.2, and partial pressure of oxygen (PO2) of 51.6, blood pressure of 59/39 mm Hg, and tachypneic, and was, therefore, intubated and started on vasopressors. The physical exam was unremarkable except for bilateral rales with decreased breath sounds. Initial labs were significant for thrombocytopenia, severe hypokalemia, elevated creatinine, troponin, and creatine kinase. Urine toxicology was positive for benzodiazepines, barbiturates, and cannabinoids.

**Figure 1:**
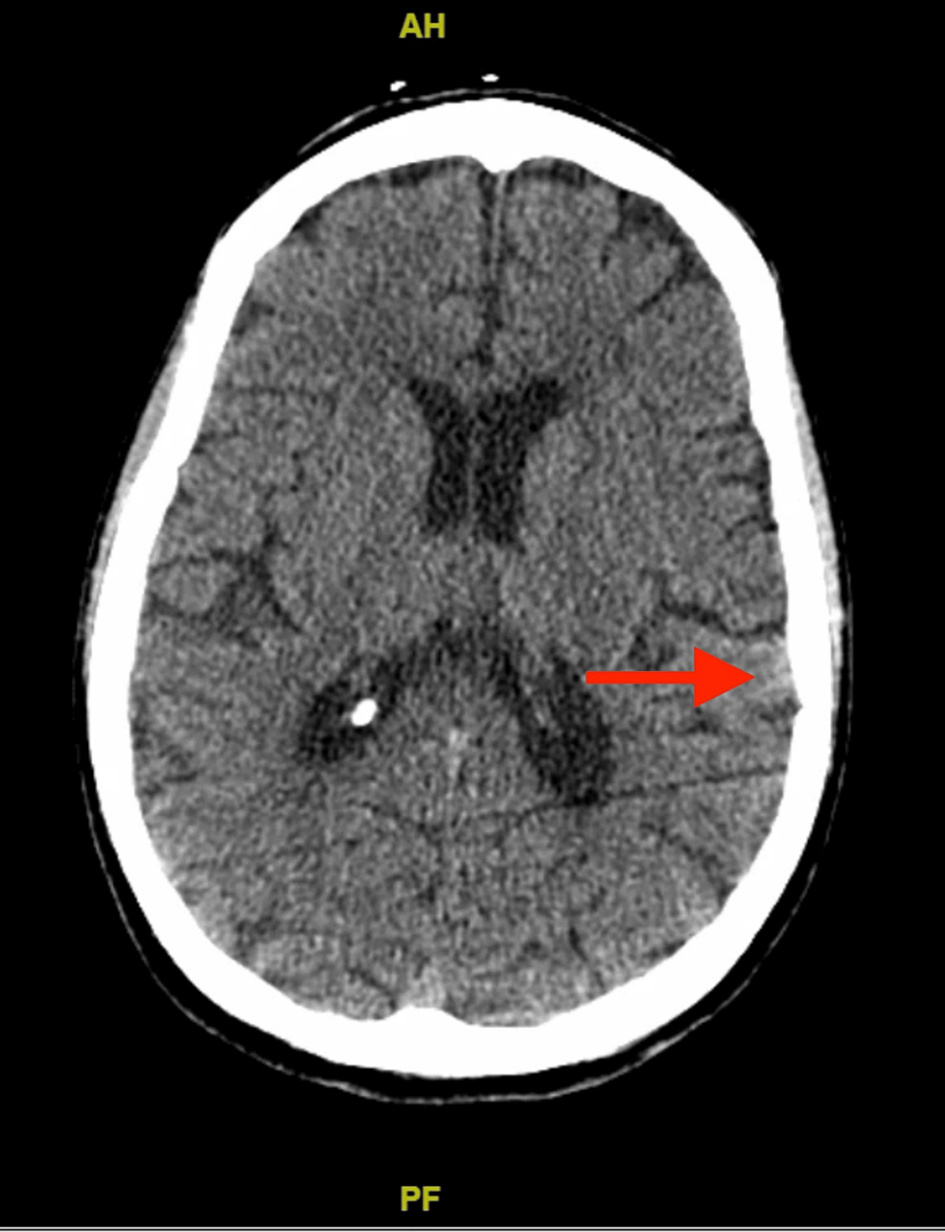
CT scan of the head showing mild chronic microvascular ischemic changes (red arrow).

**Figure 2:**
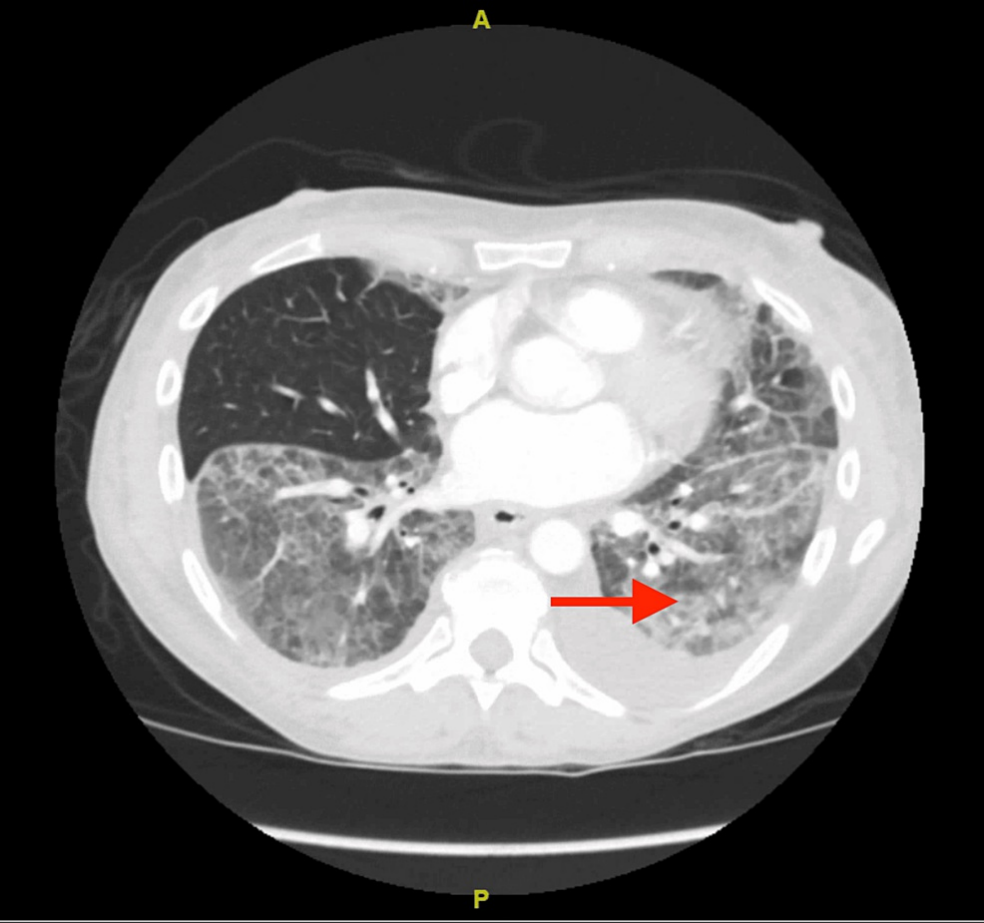
CT angiography of the chest showing bilateral ground glass opacities (red arrow) with sparing of the middle lobe.

**Figure 3:**
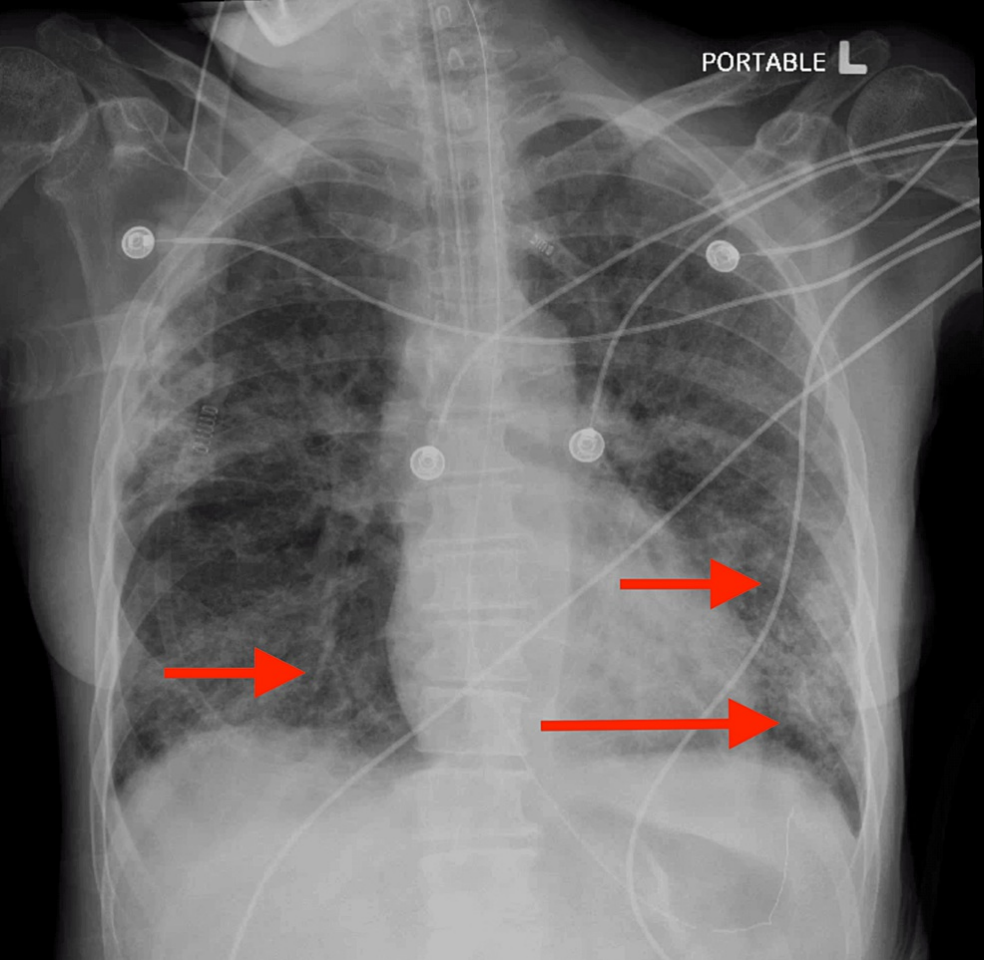
X-ray chest showing patchy opacities (red arrows).

A computed tomography (CT) scan of the head showed mild chronic microvascular ischemic changes and bilateral optic nerve tortuosity, as shown in Figure 1. CT angiography (CTA) of the chest showed bilateral ground glass opacities with sparing of the right lower lobe and distal esophageal thickening, as shown in Figure 2. X-ray chest was done, showing patchy opacities as shown in Figure 3. The patient was started on levetiracetam for seizures by Neurology. The echocardiogram showed an ejection fraction of 69.33% with grade 1 diastolic dysfunction. Cardiology was consulted for elevated troponin, deemed secondary to demand ischemia. She was stabilized in the ER and transferred to the intensive care unit (ICU). Broad-spectrum antibiotics were initiated for septic shock and bilateral lung infiltrates. An electroencephalogram showed no evidence of epileptiform activity, although they may have been suppressed as the patient was receiving propofol earlier.

On day two, a bronchoscopy was done, which showed bilateral mucopurulent secretions. Hematology was consulted for thrombocytopenia. Their impression was that thrombocytopenia may be secondary to the toxic effects of alcohol on the bone marrow but more likely multifactorial. Ultrasound of the abdomen was recommended to evaluate for hypersplenism, which was negative. She was successfully extubated on the third day of admission. Respiratory and urine cultures grew *Escherichia coli*. She later started having diarrhea and was started on oral vancomycin until *Clostridium difficile* infection was ruled out. Thrombocytopenia and hypokalemia were later resolved. Outpatient gastroenterology follow-up was recommended for esophageal thickening.

On day 11 of hospitalization, the patient was noted to be short of breath. She was started on empiric enoxaparin for suspected pulmonary embolism. She had to be re-intubated and was transferred to the ICU. Repeat chest CTA showed interval development of bilateral patchy ground glass opacities in the upper lobes and extensive interstitial opacities and ground glass opacities in the left lower lobe. The infiltrate and opacities ultimately spared the right lower lobe. To further investigate the potential vascular cause of the ARDS with lobar sparing, we considered conducting a ventilation-perfusion (V/Q) lung nuclear scanning and angiography. These diagnostic tests are crucial in evaluating the blood flow and airflow in the lungs. They could have provided valuable insights into potential vascular abnormalities contributing to the patient's condition. However, the patient declined to undergo these procedures, limiting our ability to rule out certain vascular conditions. Antibiotics and steroids were resumed. Four days later, she was extubated once again. Post extubation, the patient developed delirium. Psychosomatic Medicine was consulted and recommended divalproex, haloperidol, memantine, and melatonin. After that, the patient was once again transferred to a medical floor. Infectious Disease was consulted for the final antibiotic duration, and they recommended stopping all antibiotics as the patient had received enough vancomycin and meropenem. She was later discharged back to her nursing home.

## Discussion

In the 1960s, ARDS was initially characterized as an acute onset of hypoxia, dyspnea, and decreased lung compliance triggered by various stimuli, which did not improve with standard respiratory interventions [6]. For a brief period, it was termed "adult respiratory distress syndrome" as it mimicked infant respiratory distress syndrome; later, it was renamed "acute respiratory distress syndrome" [6]. The incidence of ARDS in the United States ranges from 64.2 to 78.9 cases per 100,000 person-years [7]. It is triggered by a variety of factors of which sepsis accounts for 32% of ARDS cases and is associated with increased severity and higher mortality rates [7,8]. 

The presented case displays a rare and intriguing manifestation of ARDS characterized by sparing the right middle lobe. This presentation challenges the conventional diagnostic and therapeutic approach. The underlying clinical significance of this presentation shows the heterogeneity of ARDS, and understanding such variations is crucial for accurate diagnosis and appropriate patient management. The pathophysiological mechanisms underlying lobar sparing need to be completed, and an adequate number of cases are present in the literature to make a proper assumption [9,10]. Typically, patients with ARDS have bilateral and diffuse lung involvement due to the broad spread of inflammatory markers [8]. There is no indication in the pathophysiology of ARDS, which may suggest an opportunity for lobar sparing.

Classical clinical manifestations of ARDS involve patient presentation of dyspnea and hypoxemia, which gets progressively worse within 672 hours of the inciting event [9]. Many times, the patient may require intubation and ICU-level care at this moment. History of recent exposure to infectious agents and environmental toxins is crucial in understanding the underlying cause of ARDS, which is generally apparent in the case of pneumonia and sepsis [9]. The patient in this report had an extensive past medical history, with recent occurrences of syncope, seizures, and septic shock. As mentioned earlier, respiratory and urine cultures at the time grew *E. coli*. Cystitis or seizures could be a possible etiology of the presenting ARDS. However, no known deviation from the pathophysiology can explain the lobar sparing.

Along with the symptoms above, a patient presenting with ARDS can also show systemic signs such as cyanosis, altered mental status, and low oxygen saturation despite being on 100% oxygen [10]. Bibasilar rales auscultated throughout the chest are significant physical exam findings in these patients [10]. The patient in this case report also presented with these symptoms of cyanosis and low oxygen saturation, requiring intubation and ICU-level care.

Diagnosis of ARDS is based on various factors; the symptoms need to be acute in onset, and there should be evidence of bilateral lung infiltrates on a chest x-ray or a CT scan [11]. Additionally, the patient should have a partial pressure of oxygen in arterial blood (PaO2)/fraction of inspiratory oxygen concentration (FiO2) ratio of less than 300 mmHg [12]. The severity of this syndrome is classified as mild, moderate, and severe based on the PaO2/FiO2 ratio [12]. Additionally, it is essential to note the left ventricular function status in the patient as it is crucial to rule out cardiological pathologies such as congestive heart failure [13]. This assessment can be completed using an echocardiogram. Per these criteria, the patient, in this case, had a CTA done for pulmonary emboli protocol, which displayed bilateral upper and lower lobe patchy ground glass opacities with subsegmental atelectasis in the left lower lobe as shown in Figure 2. Furthermore, an echocardiogram showed an ejection fraction of 69.33%, and no significant findings suggested a cardiovascular cause.

It is important to note that ARDS due to its causative factor frequently leads to concurrent multi-organ failure, particularly affecting renal, hepatic, and hematopoietic systems. Therefore, getting testing for complete blood count (CBC), comprehensive metabolic panel (CMP), serum magnesium, serum ionized calcium, phosphorus levels, blood lactate level, coagulation panel, troponin, cardiac enzymes, and CKMB [14,15]. V/Q lung nuclear scanning can point out vascular abnormalities that could potentially be implicated in this case including: (i) Interruption or absence of a main pulmonary artery. This condition can disrupt normal blood flow to the lungs, potentially contributing to ARDS; (ii) Anomalous origin of the left pulmonary artery from the right. This rare congenital defect can cause a variety of complications, including ARDS; (iii) Anomalous pulmonary venous drainage (partial or complete). This condition, where one or more pulmonary veins drain into the right atrium instead of the left, can lead to a variety of symptoms and complications; (iv) Pulmonary arteriovenous malformation. This abnormal connection between the pulmonary artery and vein can result in reduced oxygen levels in the blood, potentially leading to ARDS [15-17].

The primary treatment and management for ARDS patients is supportive care aimed at reducing the shunt fraction, increasing oxygen delivery, decreasing oxygen consumption, and avoiding additional lung triggers. As with this patient, mechanical ventilation and measures such as diuretics are required to minimize fluid overload chances. During mechanical ventilation, it is crucial to watch for barotrauma, volutrauma, and atelectrauma [15-17]. There is a mechanical ventilator setting recommended by the National Institutes of Health (NIH)-National Heart, Lung, and Blood Institute (NHLBI) ARDS Clinical Network Mechanical Ventilation Protocol; it suggests tidal volume should be 4-8 mL/kg of ideal body weight, respiratory rate up to 35 bpm, SpO2 should be 88-95%, plateau pressure less than 30 cmH2O, pH goal should be 7.30-7.45, and inspiratory-to-expiratory time ratio less than 1. Although the current patient presented with a unique presentation of ARDS, she was given a similar therapeutic approach with the goal of effective lung alveolar recruitment [18-20].

## Conclusions

This case report presents a unique presentation of ARDS with the sparing of the right middle lobe. It conveys the message to clinicians to maintain a high index of suspicion of ARDS, even in cases with atypical radiographic findings. Without the ability to perform the V/Q scan, our capacity to conclusively determine a vascular cause for this patient's ARDS with lobar sparing was limited. Future patient cooperation in diagnostic procedures will be crucial in ruling out potential vascular causes and guiding appropriate treatment strategies. The exact mechanism of the lobar sparing has yet to be understood; it shows the importance of a comprehensive diagnostic evaluation, which would allow for an individualized management plan. Continued research is needed in this area to assess the heterogeneity of ARDS presentation, which can eventually identify potential prognostic implications. Additionally, this case serves as a reminder of the importance of a multidisciplinary approach with effective collaboration between clinicians, critical care specialists, and radiologists. All in all, this would lead to earlier recognition and prompt initiation of interventions, ultimately improving outcomes for patients with atypical presentation of ARDS.
